# Safety, tolerability, pharmacokinetics, and pharmacodynamics of BI 685509, a soluble guanylyl cyclase activator, in healthy volunteers: Results from two randomized controlled trials

**DOI:** 10.1007/s00210-024-03165-w

**Published:** 2024-05-24

**Authors:** Diane Wong, Friedeborg Seitz, Verena Bauer, Thomas Giessmann, Friedrich Schulze

**Affiliations:** 1 Translational Medicine and Clinical Pharmacology, Boehringer Ingelheim Pharmaceuticals, Inc., Ridgefield, CT 06877 USA; 2 CRS Clinical Research Services Mannheim GmbH, Mannheim, Germany; 3 Boehringer Ingelheim Pharma GmbH & Co. KG, Biberach an Der Riss, Germany; 4 Boehringer Ingelheim Pharma GmbH & Co. KG, Ingelheim, Germany

**Keywords:** Cyclic guanosine monophosphate signaling, Pharmacodynamics, Pharmacokinetics, Soluble guanylate cyclase activator, Vasodilatory-stimulated phosphoprotein

## Abstract

This study evaluated the safety, tolerability, pharmacokinetics, and pharmacodynamics of BI 685509 after oral single rising doses (SRDs) or multiple rising doses (MRDs) in healthy volunteers. In the SRD trial (NCT02694354; February 29, 2016), within each of the three dose groups (DGs), six subjects received BI 685509 (1.0, 2.5, or 5.0 mg) and two received placebo (*N* = 24). In the MRD trial (NCT03116906; April 17, 2017), within each of the five DGs, nine subjects received BI 685509 (uptitrated to 1 mg once daily [qd; DG1], 2.5 mg twice daily [DG2], 5.0 mg qd [DG3]; 3.0 mg three times daily [tid; DG4] or 4.0 mg tid [DG5]) and three received placebo, for 14–17 days (*N* = 60). In the SRD trial, 7/24 subjects (29.2%) had ≥ 1 adverse event (AE), most frequently orthostatic dysregulation (*n* = 4). In the MRD trial, 26/45 subjects (57.8%) receiving BI 685509 had ≥ 1 AE, most frequently orthostatic dysregulation and fatigue (each *n* = 12). Tolerance development led to a marked decrease in orthostatic dysregulation events (DG3). BI 685509 was rapidly absorbed after oral administration, and exposure increased in a dose-proportional manner after single doses. Multiple dosing resulted in near–dose-proportional increase in exposure and limited accumulation. BI 685509 pharmacokinetics appeared linear with time; steady state occurred 3–5 days after each multiple-dosing period. Increased plasma cyclic guanosine monophosphate and decreased blood pressure followed by a compensatory increase in heart rate indicated target engagement. BI 685509 was generally well tolerated; orthostatic dysregulation may be appropriately countered by careful uptitration.

**Supplementary Information:**

The online version contains supplementary material available at 10.1007/s00210-024-03165-w.

## Introduction

Soluble guanylyl cyclase (sGC), an important component of the vasoprotective nitric oxide (NO)–sGC–cyclic guanosine monophosphate (cGMP) signaling pathway, is a receptor for NO (Budworth et al. 1999), a gaseous signaling molecule involved in several important physiologic processes, including vasodilation and vascular permeability (Hollenberg and Cinel 2009; Horst and Marletta 2018). sGC activation by NO leads to formation of cGMP from guanosine triphosphate (Budworth et al. 1999; Sandner and Stasch 2017; Wobst et al. 2015). cGMP then acts on downstream effectors, such as cGMP-regulated protein kinases and ion-gated channels (Derbyshire and Marletta 2012), to enhance vasodilation and inhibit smooth muscle cell proliferation, leukocyte recruitment, platelet aggregation, and vascular remodeling (Wobst et al. 2015; Sandner et al. 2021).

NO–sGC–cGMP pathway dysfunction has been linked to hypertension, clinically significant portal hypertension, heart failure, and chronic kidney disease (Sandner et al. 2021; Wobst et al. 2015; Morales-Ruiz et al. 2015; Langer and Shah 2006); moreover, nitrates (as a source of NO) play important clinical roles in the treatment of coronary heart disease (Giuseppe et al. 2015; Collet et al. 2021). Animal models of cirrhosis show reduced cGMP production (Schaffner et al. 2018), and hepatic levels of NO are reduced in people with cirrhosis, potentially contributing to the development of portal hypertension (Sarela et al. 1999). Activated hepatic stellate cells take on contractile characteristics, reducing portal blood flow by constricting sinusoids in the cirrhotic liver (Rockey et al. 1998; Elpek 2014; Thimgan and Yee 1999), and chronically activated hepatic stellate cells differentiate into myofibroblasts, which produce collagen and other extracellular matrix proteins, leading to fibrosis and tissue remodeling (Elpek 2014; Hall et al. 2019). In the renal vasculature, sGC is widely expressed and plays important roles in renal perfusion and renin release. Furthermore, chronic dysregulation of the NO–sGC–cGMP pathway can lead to hypertension (via glomerular capillaries), damage to glomeruli, and proteinuria.

Therefore, regulation of NO–sGC–cGMP signaling using sGC modulators, has been investigated for the treatment of hypertension and heart failure. Cinaciguat was the first NO-independent sGC activator that entered development for the treatment of people with acute decompensated heart failure; however, the development of cinaciguat was discontinued in 2013 because of the frequent occurrence of hypotension, in part due to the considerable effect of cinaciguat on the oxidized form of sGC and to the rapid “on–off” effect of cinaciguat infusion (Erdmann et al. 2013; Sawabe et al. 2019). Riociguat, a direct, NO-independent sGC stimulator marketed for the treatment of various forms of pulmonary arterial hypertension, also indirectly enhances sGC sensitivity to NO; however, because of considerable interindividual variability in riociguat exposure, a specifically tailored dosage scheme, composed of careful uptitration with three-times-daily (tid) dosing up to the highest tolerated dose for each patient, is needed prior to treatment initiation (Frey et al. 2018). Vericiguat, another NO-independent sGC stimulator, is approved in some countries for the treatment of people with heart failure and reduced ejection fraction (Markham and Duggan 2021); however, in the Phase III VICTORIA study, despite careful uptitration guided by an evaluation of blood pressure (BP) and clinical symptoms, 15.4% of vericiguat-treated participants developed hypotension, including 9.1% of vericiguat-treated participants who had symptomatic hypotension (Armstrong et al. 2020). To date, the use and development of sGC modulators has been limited by the incidence of hypotension, creating an unmet need for new sGC modulators with improved safety and tolerability profiles. Careful uptitration of these sGC modulators may help to further improve their tolerability.

Avenciguat (BI 685509) is a novel, potent sGC activator. In preclinical studies, BI 685509 reduced portal pressure and intrahepatic vascular resistance (i.e., reduced portal hypertension) and demonstrated antifibrotic properties in a bile duct ligation model of cirrhotic rats (unpublished data); BI 685509 also improved hepatic and extrahepatic cirrhosis in a thioacetamide-treated rat model of liver fibrosis and portal hypertension (unpublished data) and dose-dependently reduced the progression of diabetic nephropathy in ZSF1 obese male rats (Reinhart et al. 2023). The primary objectives of the two Phase I studies presented here were to characterize the safety and tolerability of BI 685509 in healthy volunteers after oral administration of BI 685509 in single rising doses (SRDs; 1.0, 2.5, and 5.0 mg) and multiple rising doses (MRDs; five different dosage regimens). Secondary objectives were exploration of the pharmacokinetics (PK) and pharmacodynamics (PD) of BI 685509.

## Methods

### Study ethics

The protocols of both studies were reviewed and received a favorable opinion from the Ethics Committee of the Medical Association (Ethikkommission der Landesaerztekammer) of Baden-Wuerttemberg, Stuttgart, Germany. Subsequently, the Federal Institute for Drugs and Medical Devices (Bundesinstitut für Arzneimittel und Medizinprodukte, Bonn, Germany) reviewed and approved both studies. Both studies were conducted in accordance with the principles of the Declaration of Helsinki, the International Conference on Harmonisation – Good Clinical Practice, applicable regulatory requirements, and standard operating procedures of the trial sponsor. All subjects provided written informed consent prior to participation.

### Study design and subjects

#### SRD trial

**Fig. 1:**
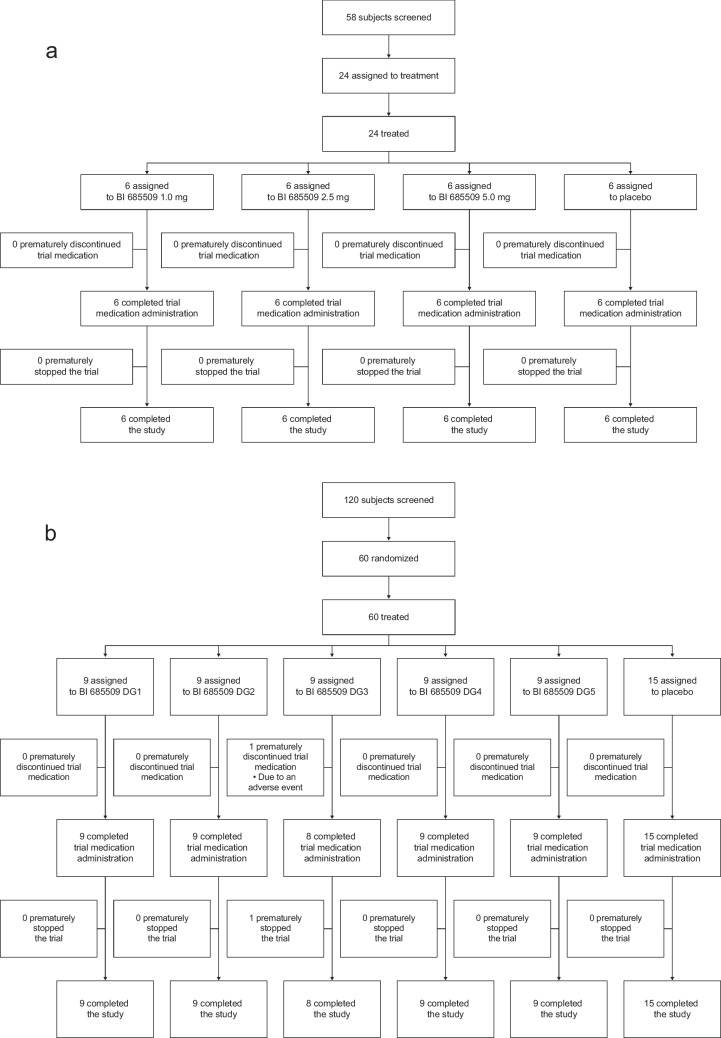
Subject disposition in the (**a**) SRD trial, (**b**) MRD trial. *DG* dose group, *MRD* multiple rising dose, *SRD* single rising dose

The SRD trial was partially randomized, placebo controlled, parallel group, and single blind, conducted at the Boehringer Ingelheim investigational site in Biberach an der Riss, Germany. Within each dose group (DG), six subjects received active treatment and two received placebo (Fig. 1a). In total, 24 healthy men aged 18–50 years with a body mass index (BMI) of 18.5 to < 30 kg/m^2^ received single oral doses of BI 685509 powder for oral solution (PfOS) 1.0 mg (*n* = 6), 2.5 mg (*n* = 6), or 5.0 mg (*n* = 6), or placebo (*n* = 6), with 240 mL of water after an overnight fast of ≥ 10 h. For the SRD and MRD studies, full details of the inclusion/exclusion criteria, stopping criteria, and additional study procedures are described in the Supplementary Information.

#### MRD trial

The MRD trial was randomized, placebo controlled, parallel group, and single blind, conducted at the Clinical Research Services site in Mannheim, Germany. Within each of five DGs, nine subjects received active treatment and three received placebo (overall: BI 685509, *n* = 45; placebo, *n* = 15; Fig. 1b). In total, 60 healthy male subjects aged 18–50 years with a BMI of 18.5 to < 30 kg/m^2^ received the following dosing regimens for 14 days (Table 1): oral doses of BI 685509 (administered as tablets) uptitrated to a total of 1.0 mg/day (*n* = 9; DG1), 5.0 mg/day (*n* = 9; DG2), 5.0 mg/day (*n* = 9; DG3), 9.0 mg/day (*n* = 9; DG4), or 12.0 mg/day (*n* = 9; DG5), or placebo (*n* = 15). Subjects in DG2 received 2.5 mg twice daily (bid) from day 11 to 17; those in DG3 received 5.0 mg once daily (qd); throughout the trial, in DG4 and DG5, BI 685509 was administered on a tid schedule (up to maximum total daily doses of 3.0 mg tid [DG4] and 4.0 mg tid [DG5]). Each dose was administered with 240 mL of water, and each morning dose was administered after an overnight fast of ≥ 10 h.

**Table 1:** BI 685509 uptitration schedules (MRD trial)

BI 685509 DG	DG1 (*n* = 9)	DG2 (*n* = 9)	DG3 (*n* = 9)
Total daily dose, mg	0.5/1.0	2.5/5.0	5.0
Single dose on day 1, mg	0.5	2.5	5.0
Dose on days 4–10 (7 days), mg	0.5 qd	2.5 qd	5.0 qd
Dose on days 11–17 (7 days), mg	1.0 qd	2.5 bid^a^	5.0 qd
BI 685509 DG	DG4 (*n* = 9)	DG5^b^ (*n* = 9)	
Total daily dose, mg	3.0/6.0/9.0	3.0/6.0/12.0	
Dose on days 1–3 (3 days), mg	1.0 tid	1.0 tid	
Dose on days 4–7 (4 days), mg	2.0 tid	2.0 tid	
Dose on days 8–14 (7 days), mg	3.0 tid	4.0 tid	

^a^Subjects received only one dose on day 17

^b^A sixth DG was originally planned; however, this DG was canceled because of severe orthostatic dysregulation on the last day of dosing in DG5

*bid* twice daily; *DG* dose group; *MRD* multiple rising dose; *qd* once daily; *tid* three times daily

### Randomization and blinding

#### SRD and MRD trials

Subjects were assigned to DGs based on their temporal availability. Subjects were randomized within each DG in a 3:1 allocation ratio (in the SRD trial, only the second cohort within each DG was randomized), with a block size of 4. The test compounds administered (BI 685509 or placebo) were blinded to the subjects only; the dose level was known to subjects. In the electrocardiogram laboratory, staff were blinded to the study treatment and electrocardiogram recording date and time.

Boehringer Ingelheim arranged for the randomization, enrollment, and intervention assignment of subjects in the trials. The randomization list was generated using a validated system, which involved a pseudo-random number generator and a supplied seed number so that the resulting allocation was reproducible and non-predictable. The randomization list of trial subject numbers was provided to the trial sites in advance of treatment initiation. Subjects were allocated to trial subject numbers by drawing lots, before the first administration of trial medication. For this purpose, the subjects were allocated to a trial subject number by drawing lots. When a trial subject number had been assigned, it could not be reassigned to any other subject.

### Outcomes

#### SRD trial

The primary endpoint was the number of subjects with drug-related adverse events (AEs), coded using the Medical Dictionary for Regulatory Activities (MedDRA) version 19.0. PK endpoints included the peak plasma BI 685509 concentration (C_max_) and the area under the plasma BI 685509 concentration–time curve (AUC) over the time interval from 0 extrapolated to infinity (AUC_0–∞_); as AUC_0–∞_ could not be determined, dose proportionality was added as a post hoc analysis for the parameter AUC_0–24_. PD endpoints included direct target-engagement markers (plasma cGMP levels), indirect target engagement markers (phosphorylated vasodilatory-stimulated phosphoprotein [p-VASP]/vasodilatory-stimulated phosphoprotein [VASP] ratio in platelets), and changes from baseline in heart rate (HR) and BP as direct markers of on-target cardiovascular effects.

#### MRD trial

The primary endpoint was the number of subjects with drug-related AEs, coded using the MedDRA version 21.0. PK endpoints included C_max_ and AUC over a uniform dosing interval τ after administration of the first dose (AUC_τ,1_; for DG1–3 on day 1); and C_max_ at steady state (C_max,ss_) and AUC at steady state over a uniform dosing interval τ (AUC_τ,ss_; for DG1–3 on days 10 and 17). PD endpoints included plasma and urinary cGMP levels (the latter standardized to urinary creatinine levels) and changes from baseline in HR.

### Safety analyses

Evaluation of safety was based on AEs (including clinically relevant findings from a physical examination), safety laboratory tests (hematology, clinical chemistry, and urinalysis), 12-lead electrocardiogram (ECG) (CardioSoft EKG System; GE Medical Systems, Freiburg, Germany), vital signs (BP, HR; Dinamap Pro 100; GE Medical Systems), mean arterial pressure, and orthostatic testing.

The term “orthostatic dysregulation” was used to describe symptomatic AEs that occurred during orthostatic testing; typical symptoms of orthostatic dysregulation are dizziness, diaphoresis, tachycardia (HR > 100 bpm), and fainting (which is reflected in the assessment of AE intensity). In the SRD study, orthostatic testing comprised four measurements: in the supine position; immediately after standing up; after 2 min in a standing position; and after 2 min of walking around. Testing was performed 30 min, 1 h, 2 h, and 3 h after study drug administration. In the MRD study, orthostatic testing was performed on days 1, 10, and 17 at 30 min, 1 h, and 2 h after study drug administration (DG1–3) and on day 1 and day 4 (1 h after morning dose), day 8 (1 h after each dose), and day 14 at 30 min, 1 h, and 2 h after morning dose (DG4 and DG5), with three measurements performed: in the supine position; immediately after standing up; and after 3 min in a standing position. A baseline measurement was performed in the morning of each trial day with planned orthostatic testing.

Hepatic injury was an AE of special interest and was defined as an elevation of aspartate transaminase (AST) and/or alanine transaminase (ALT) ≥ 3-fold upper limit of normal (ULN) combined with an elevation of total bilirubin ≥ 2-fold ULN measured in the same blood sample, and/or marked peak aminotransferase (ALT and/or AST) elevations ≥ 10-fold ULN.

### PK and PD analyses

For the PK analysis and quantification of BI 685509 plasma concentrations in both studies, 2.7 mL of blood was taken from an antecubital or forearm vein into a tripotassium ethylenediaminetetraacetic acid (K_3_-EDTA) anticoagulant blood-drawing tube at regular intervals for up to 24 h after drug dosing (SRD study) or over 20 days (MRD study). Urine samples for PK purposes were collected only from subjects in the 2.5 mg DG; a blank urine sample was collected before administration of trial medication, and urine was then collected at time points within 0–4 h, 4–8 h, 8–12 h, and 12–24 h of drug dosing in the SRD study and at pre-defined timepoints on day 1–4, 10–11 and 17–20 in the MRD study.

BI 685509 concentrations in plasma and urine were determined by a validated liquid chromatography tandem mass spectrometry assay. For the PD analysis and quantification of VASP and p-VASP in platelets (SRD study), 5.0 mL of blood was taken at regular intervals for up to 8 h after drug dosing. For quantification of cGMP in plasma, 2.7 mL of blood was taken into a K_3_-EDTA anticoagulant blood-drawing tube at prespecified times within 0.5, 1, 2, and 4 h of drug dosing (SRD study) and on days 1, 10–11, and 17 (MRD study). All PD analyses were exploratory only.

PK parameters were calculated by noncompartmental analysis using Phoenix® WinNonlin® software (version 6.3; Pharsight Corporation, Mountain View, CA, USA). Further methods for PK/PD endpoints of interest are described in the Supplementary Information.

### Statistical analyses

In both trials, the planned sample size was not based on a power calculation. The SRD trial planned to include 24 subjects (eight subjects per DG: six receiving active treatment and two receiving placebo, commonly used in SRD studies of this type and generally considered adequate for the exploratory evaluation of single-dose safety and PK) (Broom 1990). The MRD trial planned to include 72 subjects: 12 subjects per dose-escalation scheme (nine receiving active treatment and three receiving placebo, as this is considered appropriate to detect differences between dosage schedules and for the exploratory evaluation of multiple-dose safety and PK) (Broom 1990). DG6 was canceled because of severe orthostatic intolerance in one subject on the last dosing day of DG5; nonetheless, the trial was completed according to the clinical trial protocol. Descriptive statistics were calculated for all endpoints.

In the SRD trial, cGMP in plasma and the ratios of p-VASP and VASP in platelets were reported and analyzed as a percentage change from baseline over time using a repeated-measures model, using “time” and “treatment” and the interaction term “treatment x time” as fixed effects. In the MRD trial, descriptive statistics, and plots over time were provided for cGMP.

Dose proportionality was evaluated using a linear regression model applied to log-transformed data for PK endpoints in both studies (see Supplementary Information for further details). A two-sided 95% confidence interval (CI) for the slope (β) was computed; perfect dose proportionality would correspond to a slope of 1. The linearity index was calculated after multiple oral administrations based on AUC_τ,ss_ and AUC_0–∞_ in the MRD trial. Pairwise comparison of log-transformed differences permitted the calculation of two-sided 95% CIs. Back-transformed point estimates then represented an estimate of the linearity index (with perfect linearity indicated by a value of 1).

### Trial registration

The SRD and MRD trials were registered with ClinicalTrials.gov: NCT02694354 (posted on February 29, 2016) and NCT03116906 (posted on April 17, 2017), respectively.

## Results

### Demographics

In the SRD trial, all 24 subjects were White men, and all completed the study observation period. Mean age (standard deviation [SD]) of subjects was 35.8 (8.5) years; mean (SD) BMI was 25.6 (1.8) kg/m^2^ (Table 2).

**Table 2:** Baseline characteristics and demographic data in the SRD and MRD trials

Characteristic/Demographic	SRD trial					MRD trial						
	BI 685509 1.0 mg (*n* = 6)	BI 685509 2.5 mg (*n* = 6)	BI 685509 5.0 mg (*n* = 6)	Placebo (*n* = 6)	Total (*N* = 24)	BI 685509 DG1 (*n* = 9)	BI 685509 DG2 (*n* = 9)	BI 685509 DG3 (*n* = 9)	BI 685509 DG4 (*n* = 9)	BI 685509 DG5 (*n* = 9)	Placebo (*n* = 15)	Total (*N* = 60)
Sex, *n* (%)												
Male	6 (100)	6 (100)	6 (100)	6 (100)	24 (100)	9 (100)	9 (100)	9 (100)	9 (100)	9 (100)	15 (100)	60 (100)
Female	0	0	0	0	0	0	0	0	0	0	0	0
Race, *n* (%)												
Asian	0	0	0	0	0	0	0	0	0	1 (11.1)	0	1 (1.7)
Black or African American	0	0	0	0	0	0	0	1 (11.1)	0	1 (11.1)	0	2 (3.3)
White	6 (100)	6 (100)	6 (100)	6 (100)	24 (100)	9 (100)	9 (100)	8 (88.9)	9 (100)	7 (77.8)	15 (100)	57 (95.0)
Age, years, mean (SD)	37.7 (8.4)	38.2 (6.7)	35.8 (9.2)	31.5 (9.8)	35.8 (8.5)	38.6 (11.2)	39.0 (7.8)	39.0 (9.2)	43.1 (6.2)	41.1 (8.7)	–	38.7 (9.0)
Height, cm, mean (SD)	179.3 (7.1)	177.7 (5.2)	184.3 (7.3)	173.3 (6.2)	178.7 (7.3)	180.7 (8.3)	179.7 (4.3)	175.9 (6.1)	178.8 (6.6)	179.0 (9.1)	–	178.8 (6.6)
Body weight, kg, mean (SD)	82.5 (8.4)	79.2 (11.4)	88.2 (8.8)	78.5 (11.0)	82.1 (10.1)	83.8 (11.5)	81.2 (8.0)	83.2 (5.4)	81.7 (10.4)	81.0 (17.7)	–	81.6 (11.2)
BMI, kg/m^2^, mean (SD)	25.6 (1.5)	25.0 (2.4)	25.9 (1.2)	26.0 (2.0)	25.6 (1.8)	25.6 (2.1)	25.2 (2.7)	26.9 (1.7)	25.6 (3.4)	25.0 (3.1)	–	25.5 (2.7)

*BMI* body mass index; *DG* dose group; *MRD* multiple rising dose; *SD* standard deviation; *SRD* single rising dose

In the MRD trial, of 60 subjects who received study medication, one (in DG3) prematurely discontinued from the trial because of severe drug-related orthostatic dysregulation, which resolved without treatment; all other subjects (*n* = 59; 98.3%) completed the trial. Overall, 57 subjects were White (95.0%), two were Black or African American (3.3%; one each in DG3 and DG5), and one was Asian (1.7%; DG5) (Table 2). Mean (SD) age was 38.7 (9.0) years and mean (SD) BMI was 25.5 (2.7) kg/m^2^.

### Safety and tolerability

In the SRD trial, seven of 24 subjects (29.2%) had ≥ 1 AE considered by the investigator to be drug related: BI 685509 2.5 mg, *n* = 1 (16.7%); BI 685509 5.0 mg, *n* = 5 (83.3%); placebo, *n* = 1 (16.7%) (Table 3). All treatment-emergent AEs (TEAEs), except for oropharyngeal pain (one placebo recipient) and flushing (one BI 685509 2.5 mg recipient), were considered to be drug related by the investigator. The most frequent drug-related AE was orthostatic dysregulation, reported in four subjects: in the 5.0 mg DG, three subjects experienced severe, symptomatic orthostatic dysregulation 30 min post dose (when plasma BI 685509 concentration was > 200 nmol/L in all affected subjects); in the 2.5 mg DG, one case of mild orthostatic dysregulation was reported 1 h post dose (when the plasma BI 685509 concentration was 150 nmol/L). Besides orthostatic dysregulation, two subjects had mild headache and one had mild nausea. No serious AEs or AEs of special interest occurred in the SRD trial.

**Table 3:** Summary of adverse events: SRD trial

AE	BI 685509 1.0 mg (*n* = 6)	BI 685509 2.5 mg (*n* = 6)	BI 685509 5.0 mg (*n* = 6)	Placebo (*n* = 6)	Total (*N* = 24)
Any TEAE, *n* (%)	0	2 (33.3)	5 (83.3)	2 (33.3)	9 (37.5)
Orthostatic dysregulation	0	1 (16.7)	3 (50.0)^b^	0	4 (16.7)
Headache	0	0	1 (16.7)	1 (16.7)	2 (8.3)
Flushing	0	1 (16.7)	0	0	1 (4.2)
Nausea	0	0	1 (16.7)	0	1 (4.2)
Oropharyngeal pain	0	0	0	1 (16.7)	1 (4.2)
Investigator-defined, drug-related AEs,^a^ n (%)	0	1 (16.7)	5 (83.3)	1 (16.7)	7 (29.2)
Severe AEs, *n* (%)	0	0	3 (50.0)	0	3 (12.5)
Serious AEs, *n* (%)	0	0	0	0	0
AEs leading to discontinuation of study drug, *n* (%)	0	0	0	0	0

^a^All TEAEs, except for flushing (*n* = 1, BI 685509 group) and oropharyngeal pain (*n* = 1, placebo group), were considered to be drug related

^b^One of these subjects had an episode of orthostatic dysregulation and an episode of asymptomatic orthostatic hypotension

*AE* adverse event; *SRD* single rising dose; *TEAE* treatment-emergent AE

Owing to the cases of severe orthostatic dysregulation, dose escalation did not progress beyond a single dose of BI 685509 5.0 mg, in line with predefined stopping criteria. Subsequently, the MRD trial tested various BI 685509 dosage schedules to determine whether multiple dosing improved tolerability (because of the development of tolerance) and whether splitting the administration of BI 685509 would permit higher total daily doses (including bid and tid dosing; Table 1).

In the MRD trial, 28 of 45 subjects (62.2%) receiving BI 685509 and 8 of 15 subjects (53.3%) receiving placebo had ≥ 1 TEAE; investigator-defined, drug-related AEs occurred in 26 of 45 subjects (57.8%) receiving BI 685509 and in none of the 15 subjects receiving placebo (Table 4). The most frequently reported drug-related AEs were orthostatic dysregulation and fatigue. Drug-related orthostatic dysregulation occurred in 12 subjects (26.7%). In DG1, no orthostatic dysregulation occurred, whereas in DG2 one subject (11.1%) experienced this AE (mild intensity) on the first treatment day. In DG3, eight subjects (88.9%) had orthostatic dysregulation between days 1 and 10, and two (25.0%) between days 11 and 17. On day 1 in DG3, orthostatic dysregulation was severe in five BI 685509-treated subjects and moderate in two subjects; of those with severe orthostatic dysregulation, one was withdrawn from treatment after day 1 because of syncope that had occurred before the first orthostatic testing. Generally, the cases of orthostatic dysregulation were closely associated with the high plasma BI 685509 concentration recorded at approximately the time of peak plasma concentration (t_max_; i.e., typically within 0.5–1.0 h post dose). Tolerance development in DG3 led to a marked decrease in orthostatic dysregulation events: on day 10, only one case of orthostatic dysregulation (severe) occurred, 30 min post dose (the plasma BI 685509 concentration was 227 nmol/L); on day 17, one subject had mild orthostatic dysregulation (< 200 nmol/L) and one had moderate orthostatic dysregulation (plasma BI 685509 concentration was 248 nmol/L at 30 min post dose). Besides orthostatic dysregulation, the most frequent drug-related AEs in DG1–3 were headache (11 subjects; mild) and fatigue (eight subjects: mild in DG1 and DG2 [four subjects], moderate in DG3 [four subjects]). In DG3, one subject each experienced drug-related asthenia (moderate intensity), ear discomfort (moderate), dizziness (mild), and nausea (mild); as a result, it was decided not to increase the dose to 5.0 mg bid.

**Table 4:** Incidence of investigator-defined, drug-related adverse events: MRD trial

AE,^a^ *n* (%)	DG1 0.5 mg qd days 1, 4–10 (*n* = 9)	DG1 1.0 mg qd days 11–17 (*n* = 9)	DG2 2.5 mg qd days 1, 4–10 (*n* = 9)	DG2 2.5 mg bid days 11–17 (*n* = 9)	DG3 5.0 mg qd days 1, 4–10 (*n* = 9)	DG3 5.0 mg qd days 11–17 (*n* = 8)	DGs 4 and 5 1 mg tid days 1–3 (*n* = 18)	DGs 4 and 5 2.0 mg tid days 4–7 (*n* = 18)	DG4 3.0 mg tid days 8–14 (*n* = 9)	DG5 4.0 mg tid days 8–14 (*n* = 9)	Total (*N* = 45)
Any AE	3 (33.3)	1 (11.1)	3 (33.3)	2 (22.2)	8 (88.9)	4 (50.0)	4 (22.2)	2 (11.1)	3 (33.3)	6 (66.7)	26 (57.8)
Orthostatic dysregulation	0	0	1 (11.1)	0	8 (88.9)	2 (25.0)	0	1 (5.6)	0	2 (22.2)	12 (26.7)
Headache	1 (11.1)	1 (11.1)	3 (33.3)	1 (11.1)	4 (44.4)	1 (12.5)	0	0	1 (11.1)	1 (11.1)	11 (24.4)
Dizziness	0	0	0	0	1 (11.1)	0	1 (5.6)	0	2 (22.2)	0	4 (8.9)
Attention disturbance	0	0	0	0	2 (22.2)	0	0	1 (5.6)	0	0	3 (6.7)
Head discomfort	0	0	1 (11.1)	0	0	0	0	0	0	0	1 (2.2)
Syncope	0	0	0	0	1 (11.1)	0	0	0	0	0	1 (2.2)
Fatigue	3 (33.3)	0	1 (11.1)	0	2 (22.2)	2 (25.0)	2 (11.1)	1 (5.6)	2 (22.2)	2 (22.2)	12 (26.7)
Asthenia	0	0	0	0	1 (11.1)	1 (12.5)	0	0	0	0	2 (4.4)
Nausea	0	0	0	0	0	1 (12.5)	0	0	1 (11.1)	1 (11.1)	3 (6.7)
Diarrhea	0	0	0	0	1 (11.1)	0	0	0	0	0	1 (2.2)
Toothache	0	0	0	1 (11.1)	0	0	0	0	0	0	1 (2.2)
Abdominal distension	0	0	0	0	0	0	1 (5.6)	0	0	0	1 (2.2)
Polyuria	0	0	0	0	0	0	2 (11.1)	0	0	1 (11.1)	3 (6.7)
Ear discomfort	0	0	0	0	1 (11.1)	1 (12.5)	0	0	0	0	1 (2.2)
Decreased appetite	0	0	0	0	0	0	1 (5.6)	0	0	0	1 (2.2)
Hyperhidrosis	0	0	0	0	0	0	0	0	0	1 (11.1)	1 (2.2)

^a^No drug-related AEs were reported in subjects who received placebo

*AE* adverse event; *bid* twice daily; *DG* dose group; *MRD* multiple rising dose; *qd* once daily; *tid* three times daily

DG4 and DG5 assessed the effects of BI 685509 dosage uptitration to tid administration. No orthostatic dysregulation occurred in DG4 (BI 685509 3.0 mg tid), indicating good orthostatic tolerance. In DG5 on day 14, one subject had mild orthostatic dysregulation and one had severe orthostatic dysregulation (30 min post dose, plasma BI 685509 concentration > 200 nmol/L); therefore, dose escalation beyond DG5 was not performed. Besides orthostatic dysregulation, the most frequent drug-related AEs in DG4 and DG5 were fatigue (seven subjects; mild intensity), dizziness (three subjects; mild), polyuria (three subjects; mild), headache (two subjects; one mild, one moderate), and nausea (two subjects; mild). As these AEs were mild (except for one case of headache), BI 685509 tolerability appeared to be improved with dosage uptitration to tid dosing (DG5) compared with 5.0 mg qd dosing (DG3).

There were no notable changes in the QT interval on 12-lead electrocardiograms in the SRD and MRD trials: none of the subjects had a new onset of QT interval (uncorrected or corrected for HR according to Fridericia’s formula) of > 500 ms or an increase from baseline in the QT interval corrected for HR according to Fridericia’s formula (QTcF) of > 60 ms. However, in the MRD study, one subject in DG1 had a maximum on-treatment change from baseline in uncorrected QT interval of > 60 ms, and one placebo recipient had a new onset of maximum on-treatment QTcF interval in the range of 450 to 480 ms. Additionally, one subject in the SRD trial (BI 685509 5.0 mg) and six subjects in the MRD trial (DG2, *n* = 1; DG3, *n* = 3; placebo, *n* = 2) had a maximum change in the QTcF interval between > 30 ms and ≤ 60 ms.

### PK results

**Fig. 2:**
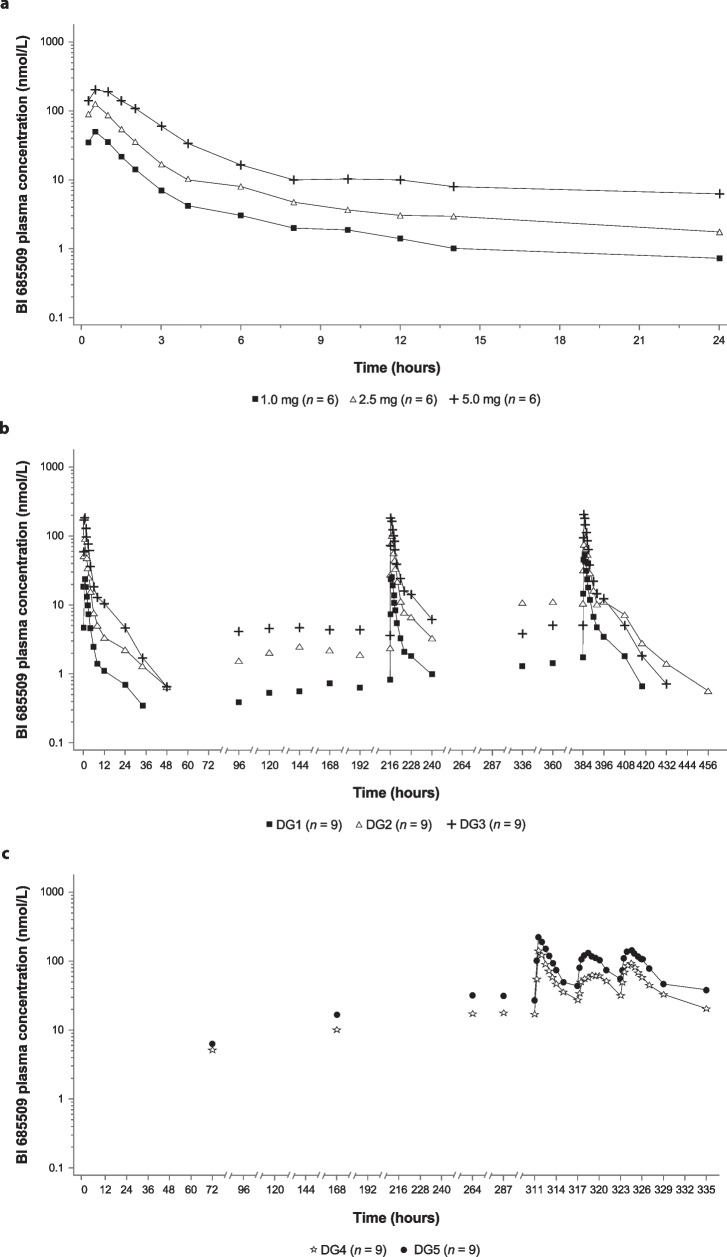
Geometric mean plasma BI 685509 concentration–time profiles (semi-logarithmic scale) (**a**) after single oral administration of BI 685509 1.0 mg, 2.5 mg, and 5.0 mg doses: SRD trial; (**b**) after single and multiple oral administration of BI 685509: MRD trial (DG1–3); (**c**) after multiple administration of BI 685509: MRD trial (DG4 and DG5). *DG* dose group, *MRD* multiple rising dose, *SRD* single rising dose

Plasma BI 685509 concentration–time profiles increased with rising doses after single oral administration (Fig. 2a): BI 685509 was rapidly absorbed, reaching peak levels at approximately 0.5 h post dose (Table 5); plasma concentrations then declined in an at least biphasic manner. BI 685509 PK were close to dose proportional (slope for C_max_ 0.9402 [95% CI 0.6775–1.2029]; AUC_0–24_ 1.1095 [95% CI 0.8051–1.4139]). As plasma samples were collected up to 24 h, which is insufficient to characterize the terminal half-life and related PK parameters, single-dose administrations were further assessed in the MRD trial.

**Table 5:** Summary of BI 685509 PK parameters: SRD trial

Parameter	BI 685509 1.0 mg (*n* = 6)		BI 685509 2.5 mg (*n* = 6)		BI 685509 5.0 mg (*n* = 6)	
	gMean	gCV (%)	gMean	gCV (%)	gMean	gCV (%)
C_max_, nmol/L	48.4	22.8	121	26.4	219	54.9
AUC_0–24_, nmol·h/L	105	32.3	258	42.6	634	51.7
t_max_,^a^ h	0.500	0.500–1.00	0.500	0.500–0.517	0.517	0.500–1.00
fe_0–24_, %	–	–	0.354	81.7	–	–
CL_R,0–24_, mL/min	–	–	0.982	50.1	–	–

^a^Values are median and range

*AUC*_*0–24*_ area under the plasma concentration–time curve from 0 to 24 h; *CL*_*R,0–24*_ renal clearance from 0 to 24 h; *C*_*max*_ peak plasma concentration; *fe*_*0–24*_ fraction excreted from 0 to 24 h; *gCV* geometric coefficient of variation; *gMean* geometric mean; *SRD* single rising dose; *t*_*max*_ time to peak plasma concentration

After single-dose oral administration of BI 685509 0.5, 2.5, or 5.0 mg on day 1 in the MRD trial, BI 685509 was rapidly absorbed, with t_max_ reaching approximately 0.5–1.0 h post dose. Concentrations then declined in an at least biphasic manner, consistent with the SRD trial results. After multiple-dose oral administration of BI 685509, the plasma concentration–time profiles were similar for all DGs and were consistent with single-dose PK profiles (Fig. 2b, c). Low-to-high interindividual variability of the plasma concentration–time profiles was observed (the geometric coefficient of variation ranged from 11.2% to 268%).

Overall, the PK of multiple doses of BI 685509 were consistent with the single-dose results (Table 6). After single and multiple oral administrations, exposure (C_max_ and AUC values) increased with increasing dose. Apparent clearance (CL/F and CL/F_ss_) ranged from 147 to 208 mL/min, and apparent volumes of distribution (V_z_/F and V_z_/F_ss_) were 127 to 257 L. Terminal half-life (t_½_ and t_½,ss_) ranged from 9.04 to 14.5 h across DGs. BI 685509 PK were dose proportional or close to dose proportional after single and multiple dosing (Supplementary Table S1). After multiple dosing, limited accumulation of BI 685509 was observed with qd or bid administration (R_A,Cmax_ 0.968–1.08; R_A,AUC_ 1.22–1.24) and the linearity index (1.03–1.09) showed that BI 685509 PK were near-linear with respect to time. Steady state appeared to be attained by approximately 3–5 days after the start of each multiple-dosing period. Renal clearance (CL_R,0–24_ and CL_R,0–τ,ss_) after single- and multiple-dose administration of BI 685509 was low (0.421–0.905 mL/min), and < 1% of the administered BI 685509 dose was excreted unchanged in urine, consistent with the SRD trial results. After uptitration and tid administration in DG4 and DG5, the total BI 685509 exposures achieved were higher than in DG1–3.

**Table 6:** Summary of BI 685509 PK parameters after oral administration of single and multiple doses: MRD trial

Parameter^a^	DG1 (*n* = 9)		DG2 (*n* = 9)		DG3 (*n* = 9)	
	gMean	gCV (%)	gMean	gCV (%)	gMean	gCV (%)
Day 1	0.5 mg		2.5 mg		5.0 mg	
AUC_0–24_, nmol·h/L	76.6	26.0	294	31.1	626	47.5
C_max_, nmol/L	24.6	23.3	113	24.5	221	50.6
t_max_,^b^ h	1.00	0.500–1.50	0.500	0.500–0.500	0.500	0.500–1.00
V_z_/F, L	171	40.7	257	54.4	163	53.5
CL/F, mL/min	162	24.2	204	33.2	208	49.4
t_½_, h	12.2	18.3	14.5	54.3	9.04	25.2
fe_0–72_, %	0.513	51.6	0.349	75.0	0.408	57.4
CL_R,0–24_, mL/min	–	–	0.800	83.1	0.893	25.5
Day 10	0.5 mg qd (from day 4)		2.5 mg qd (from day 4)		5.0 mg qd (from day 4)^c^	
AUC_0–24,ss,8_, nmol·h/L	93.5	27.0	361	24.7	700	8.98
C_max,ss,8_, nmol/L	26.6	18.6	110	15.6	216	36.9
t_max,ss,8_,^b^ h	1.00	0.500–1.00	1.00	0.500–1.50	0.500	0.500–2.50
CL/F_ss,8_, mL/min	153	27.0	198	24.7	204	8.98
fe_0–24,ss,8_, %	0.430	49.5	0.423	40.8	0.443	41.5
CL_R,0–24,ss,8_, mL/min	–	–	0.837	52.5	0.905	37.6
R_A,Cmax,8_	1.08	10.7	0.968	28.0	1.06	47.3
R_A,AUC,τ,8_	1.22	14.1	1.23	17.8	1.23	32.9
Linearity index	1.06	13.6	1.03	17.2	1.14	31.1
Day 17	1.0 mg qd (from day 11)		2.5 mg bid (from day 11)		5.0 mg qd (from day 4)^c^	
AUC_0–24,ss,15_, nmol·h/L	195	20.4	–	–	693	18.6
C_max,ss,15_, nmol/L	57.2	20.4	–	–	235	24.2
t_max,ss,15_,^b^ h	0.633	0.500–1.00	–	–	0.500	0.500–1.50
V_z_/F_ss,15_, L	127	38.2	–	–	171	31.9
CL/F_,s,15_, mL/min	147	20.4	–	–	206	18.6
t_½,ss,15_, h	10.0	28.2	–	–	9.55	18.0
fe_0–24,ss,15_, %	0.559	27.1	–	–	0.389	53.2
CL_R,0–24,ss,15_, mL/min	0.804	37.8	–	–	0.802	47.6
R_A,Cmax,15_	–	–	–	–	1.15	49.2
R_A,AUC,τ,15_	–	–	–	–	1.22	33.7
AUC_0–12,ss,21_, nmol·h/L	–	–	363	26.8	–	–
C_max,ss,21_, nmol/L	–	–	112	30.3	–	–
t_max,ss,21_,^b^ h	–	–	1.00	0.500–1.50	–	–
V_z_/F_ss,21_, L	–	–	212	32.6	–	–
CL/F_,ss,21_, mL/min	–	–	196	26.4	–	–
t_½,ss,21_, h	–	–	12.5	31.7	–	–
fe_0–12,ss,21_, %	–	–	0.482	55.2	–	–
CL_R,0–12,ss,21_, mL/min	–	–	0.949	58.4	–	–
R_A,Cmax,21_	–	–	0.992	29.0	–	–
R_A,AUC,τ,21_	–	–	1.24	19.1	–	–
Linearity index	–	–	–	–	1.13	32.8
	DG4 (*n* = 9)		DG5 (*n* = 9)			
	gMean	gCV (%)	gMean	gCV (%)		
	1.0/2.0/3.0 mg tid		1.0/2.0/4.0 mg tid			
AUC_0–24,40_,^d^ nmol·h/L	1210	26.5	1980	26.8		
C_max,40_,^d^ nmol/L	145	27.2	235	23.8		
t_max,40_,^b,d^ h	0.500	0.500–1.00	0.500	0.500–1.50		
fe_0–24,40_,^d^ %	0.261	66.7	0.241	49.1		
CL_R,0–24,40_,^d^ mL/min	0.557	46.7	0.421	50.4		

^a^Subscript numbers (e.g., C_max,ss,8_) represent the nth dose

^b^For t_max_, data shown are median and range

^c^*n* = 8

^d^On the last study day in DG4 and DG5, PK parameters determined over 0–24 h were calculated after tid administration of BI 685509, whereas C_max,40_ and t_max,40_ were determined after the first morning dose only

*AUC* area under the plasma concentration–time curve; *bid* twice daily; *CL/F* apparent clearance; *CL*_*R*_ renal clearance; *C*_*max*_ maximum plasma concentration; *DG* dose group; *fe* fraction excreted; *gCV* geometric coefficient of variation; *gMean* geometric mean; *MRD* multiple rising dose; *qd* once daily; *R*_*A,AUC*_ accumulation ratio after multiple dosing based on AUC over a uniform dosing interval expressed as the ratio of AUC at steady state and after single dose; *R*_*A,Cmax*_ accumulation ratio after multiple dosing based on C_max_ over a uniform dosing interval expressed as the ratio of C_max_ at steady state and after single dose; *ss* steady state; *t*_*½*_ terminal half-life; *tid* three times daily, *t*_*max*_ time to peak plasma concentration; *V*_*z*_*/F* apparent volume of distribution

### PD results

In the SRD trial, an increase in p-VASP/VASP ratio was observed for BI 685509 5.0 mg versus placebo at 0.5 h post dose; the ratio relative to placebo was 1.19 (95% CI 1.05–1.35) (Supplementary Fig. S1). A notable increase in plasma cGMP concentrations was observed for BI 685509 5.0 mg versus placebo at 2 h post dose; the ratio relative to placebo was 1.29 (95% CI 1.06–1.59) (Supplementary Fig. S2). In general, BP decreased after dosing with BI 685509 compared with baseline, with the largest difference compared with placebo at 1 h post dosing. BP decline was followed by a compensatory HR increase. A placebo-corrected HR change from baseline of 9.0 beats/minute (bpm; adjusted mean) was recorded at 1 h post dose in the BI 685509 2.5 mg group. For the BI 685509 5.0 mg group, placebo-corrected HR changes from baseline of 11.9 bpm and 12.6 bpm (adjusted mean) were recorded at 1 h and 2 h post dose, respectively.

In the MRD trial, the time to reach peak plasma cGMP concentration was approximately 1 h after BI 685509 administration; although there was a slight trend toward higher mean cGMP levels at higher BI 685509 doses, there was no definitive evidence of a dose-dependent effect. Generally, baseline-corrected urinary cGMP levels overlapped between BI 685509 DGs and placebo; there was no observable dose-dependent effect of urinary cGMP generation relative to increasing doses of BI 685509 (Supplementary Fig. S3 and S4). In general, BP decreased after dosing with BI 685509 compared with baseline in all DGs (Supplementary Table S2). The largest decreases were observed in DG1–3 approximately 1 h after dosing on day 1. In addition, in the MRD trial, BP decrease was followed by a compensatory HR increase. In the first hour post dose on day 1, the mean HR change from baseline appeared to be greater in DG3 versus other DGs: that is, on day 1, the greatest increase in HR was seen 30 min post dose when the mean change from baseline was + 7.7 bpm in DG3, compared with –1.1, –3.1, and + 2.7 bpm in the placebo group, DG1, and DG2, respectively.

## Discussion

Single oral doses of BI 685509 1.0 mg or 2.5 mg were generally well tolerated, as were MRDs up to 3.0 mg tid. However, BI 685509 5.0 mg (as a single dose in the SRD trial and qd in the MRD trial [DG3]) was not tolerated by healthy subjects as severe orthostatic dysregulation occurred in half of the subjects who received BI 685509 5.0 mg in the SRD trial and in six subjects treated with 5.0 mg/day (DG3; five of these events occurred on day 1, one occurred on day 10) in the MRD trial. Multiple daily dosing with BI 685509 appeared to improve cardiovascular tolerability because no orthostatic dysregulation occurred in DG4 (3 mg tid); however, one case of mild and one of severe orthostatic dysregulation (both associated with a plasma BI 685509 concentration of > 200 nmol/L) occurred on day 14 in DG5 (4.0 mg tid)*,* causing the pre-planned progression to 5.0 mg tid to be canceled. Typically, the occurrence of orthostatic dysregulation was closely linked with the high plasma BI 685509 concentration determined at approximately the t_max_ of 0.5 h to 1.0 h post dose. Reports of orthostatic dysregulation at the higher dose of BI 685509 were not unexpected due to the mechanism of action of BI 685509 as an activator of sGC, which converts guanosine triphosphate into the second messenger cGMP (Aktories et al. 2013; Krishnan et al. 2018; Theilig et al. 2001). By regulating cGMP-dependent protein kinase G (PKG), cGMP reduces intracellular calcium concentrations in smooth muscle cells, thereby mediating a vascular relaxation. The more detailed PKG1 phosphorylates the inositol 1,4,5-trisphosphate (IP_3_) receptor and inositol 1,4,5-triphosphate receptor-associated protein (IRAG), an IP_3_ receptor-associated protein. The phosphorylation of IRAG inhibits the IP_3_-mediated release of calcium from the endoplasmic reticulum. Thus, activation of sGC may result in hypotension (Aktories et al. 2013), which is a previously reported class effect of sGC modulators (Armstrong et al. 2020; Erdmann et al. 2013; Frey et al. 2018; Markham and Duggan 2021; Sawabe et al. 2019). Overall, orthostatic dysregulation was reported for 12 of 45 subjects (26.7%) receiving BI 685509 and one of 15 subjects (6.7%) receiving placebo; with the careful uptitration of the BI 685509 dose over time, orthostatic dysregulation incidence in the BI 685509 DGs was reduced because of cardiovascular adaptation.

Besides orthostatic dysregulation, the most frequent drug-related AE in the MRD trial was fatigue, which occurred in all DGs and reached moderate intensity in DG3. Generally, the variability and intensity of AEs increased from DG1 to DG3, whereas drug-related AEs were of mild intensity in DG4 and DG5, except for one case of moderate headache, thus suggesting improved tolerability associated with BI 685509 uptitration and tid dosing relative to 5.0 mg qd dosing in DG3. Overall, the safety profile of BI 685509 was consistent with the pharmacologic mode of action (sGC activation) and with observations from preclinical studies, which also revealed reduced BP after BI 685509 administration.

Our findings are similar to those observed in Phase I studies of sGC stimulators. For example, features of the PK profile of orally administered riociguat are rapid absorption, almost complete bioavailability, and dose-proportional exposure (Frey et al. 2018). Riociguat was linked to transient reductions in BP and increased HR, but was generally well tolerated: only one of 36 subjects (2.8%) experienced severe hypotension, which was also considered serious (Frey et al. 2016). The PK profile of vericiguat was characterized by non-deviation from dose proportionality and no unexpected accumulation, and oral doses of ≤ 10 mg were generally well tolerated (Boettcher et al. 2021). However, in the first-in-human study of vericiguat administered as single oral doses of 0.5 mg to 15.0 mg, drug-related headache and postural dizziness were each reported in 7.2% of vericiguat-treated subjects; three of four subjects (75%) treated with vericiguat 15 mg experienced orthostatic reactions (Boettcher et al. 2021).

In our studies, although two different formulations of BI 685509 were administered (PfOS in the SRD trial and tablets in the MRD trial), the PK results were consistent, suggesting that tablet dissolution does not impact BI 685509 absorption. In both studies, BI 685509 PK results were characterized by rapid absorption and biphasic distribution and elimination. Systemic exposure to BI 685509 increased in a dose-proportional manner after administration of single doses, and close to dose-proportional exposure was observed at steady state for the dose range tested (0.5–5.0 mg). After multiple oral administrations, BI 685509 PK appeared to be linear with time and limited accumulation was observed. Steady state appeared to be attained by approximately 3–5 days after the start of qd or bid dosing regimens. Renal excretion of BI 685509 was low. With tid versus qd or bid dosing regimens, higher total daily exposures could be achieved without substantially increasing peak plasma BI 685509 concentrations. Phosphorylation of VASP has been shown to be a marker of cGMP-dependent protein kinase activation (Waldmann et al. 1987); PD results confirmed an increase in p-VASP/VASP ratio and cGMP concentrations in the blood of subjects receiving BI 685509 versus placebo.

Our results are associated with the traditional limitations of Phase I studies, including the short duration of treatment. In addition, low subject numbers may have confounded the PD findings, particularly regarding results for direct and indirect target-engagement markers (cGMP levels and p‑VASP/VASP ratio, respectively). Nevertheless, transient reductions in BP followed by a compensatory HR increase from baseline confirmed on-target cardiovascular effects for BI 685509. However, the focus of the current study was safety and tolerability, and, based on our findings in this trial, specific PD effects of the following doses of BI 685509 (after uptitration according to individual tolerability) were evaluated in subsequent studies: 1.0 mg to 3.0 mg bid in the 1366.20 Phase Ib trial in people with mild and moderate hepatic impairment (NCT03842761) (Lawitz et al. 2023); 3.0 mg qd, or 1.0 mg or 3.0 mg tid, in the 1366.04 Phase Ib trial in people with diabetic kidney disease (NCT03165227) (Cherney et al. 2023). Doses of 2.0 mg or 3.0 mg bid (after uptitration according to individual tolerability) will be evaluated in the 1366.21 (NCT05161481) and 1366.29 (NCT05282121) Phase II studies in people with clinically significant portal hypertension. Finally, doses of 1–3 mg tid are being assessed in two Phase II studies in people with chronic kidney disease (NCT04750577; NCT04736628).

## Conclusions

The novel, potent sGC activator, BI 685509 was generally well tolerated at single oral doses of 1.0 mg and 2.5 mg, and at MRDs up to 3.0 mg tid in healthy volunteers. BI 685509 PK results were generally dose proportional and appeared linear with time. In addition, the results suggest that first-dose orthostatic dysregulation may be appropriately countered by careful initial dose selection and subsequent uptitration.

## Supplementary Information

Below is the link to the electronic supplementary material.

Supplementary file1 (DOCX 360 KB)

## Data Availability

To ensure independent interpretation of clinical study results and enable authors to fulfill their role and obligations under the ICMJE criteria, Boehringer Ingelheim grants all external authors access to relevant clinical study data. In adherence with the Boehringer Ingelheim Policy on Transparency and Publication of Clinical Study Data, scientific and medical researchers can request access to clinical study data after publication of the primary manuscript and secondary analyses in peer-reviewed journals and regulatory and reimbursement activities are completed, normally within 1 year after the marketing application has been granted by major Regulatory Authorities. Researchers should use the https://vivli.org/ link to request access to study data and visit https://www.mystudywindow.com/msw/datasharing for further information.
