# Supplementary material for: Safety, tolerability, pharmacokinetics, and pharmacodynamics of BI 685509, a soluble guanylyl cyclase activator, in healthy volunteers: Results from two randomized controlled trials
Source: Naunyn Schmiedebergs Arch Pharmacol. 2024 May 24;397(10):8101–16. doi: 10.1007/s00210-024-03165-w (PMC11449976; doi:10.1007/s00210-024-03165-w)

## Supplementary Information

# **Safety, tolerability, pharmacokinetics, and pharmacodynamics of BI 685509, a soluble guanylyl cyclase activator, in healthy volunteers: Results from two randomized controlled trials**

## Diane Wong, Friedeborg Seitz, Verena Bauer, Thomas Giessmann, and Friedrich Schulze

**Corresponding Author:**

Diane Wong, Translational Medicine and Clinical Pharmacology, Boehringer Ingelheim Pharmaceuticals, Inc., Ridgefield, CT 06877, USA.

Email: [diane.wong@boehringer-ingelheim.com](mailto:diane.wong@boehringer-ingelheim.com)

## *Methods*

#### Inclusion Criteria

1. Healthy men, according to the investigator’s assessment, based on a complete medical history, including a physical examination, vital signs (blood pressure [BP], pulse rate [PR]), 12‑lead electrocardiogram (ECG), and clinical laboratory tests
2. Age 18–50 years (inclusive)
3. Body mass index (BMI) 18.5–29.9 kg m^–2^ (inclusive)
4. Signed and dated written informed consent before admission to the trial, in accordance with Good Clinical Practice and local legislation

#### Exclusion Criteria

1. Any finding in the medical examination (including BP, PR, or ECG) deviating from normal
   and judged as clinically relevant by the investigator
2. Repeated measurement of systolic BP outside 100–140 mmHg, diastolic BP outside
   60–90 mmHg, or PR outside 50–90 beats per minute (bpm)
3. Any laboratory value outside the reference range that the investigator considered to be clinically relevant
4. Any evidence of a concomitant disease judged as clinically relevant by the investigator
5. Gastrointestinal, hepatic, renal, respiratory, cardiovascular, metabolic, immunologic,
   or hormonal disorders
6. Cholecystectomy and/or surgery of the gastrointestinal tract (except appendectomy
   and simple hernia repair) that could interfere with the pharmacokinetics (PK) of the
   trial medication
7. Diseases of the central nervous system (including but not limited to any kind of seizure
   or stroke) and other relevant neurologic or psychiatric disorders
8. History of relevant orthostatic hypotension, fainting spells, or blackouts
9. Chronic or relevant acute infections
10. History of relevant allergy or hypersensitivity (including allergy to the trial medication
    or its excipients)
11. Intake of drugs with a long half-life (>24 h) within 30 days or <10 half-lives of the respective drug before administration of trial medication
12. Within 10 days before administration of trial medication, use of drugs that might reasonably influence the trial results or that might prolong the QT/QTc interval
13. Participation in another trial where an investigational drug has been administered within 60 days before planned administration of trial medication
14. Smoker (more than 10 cigarettes or 3 cigars or 3 pipes per day)
15. Inability to refrain from smoking on specified trial days
16. Alcohol abuse (consumption of >24 g day^−1^)
17. Drug abuse or positive drug screening
18. Blood donation of >100 mL within 30 days before administration of trial medication,
    or intended blood donation during the trial
19. Intention to perform excessive physical activities within 1 week before administration of trial medication or during the trial
20. Inability to comply with dietary regimen of the trial site
21. Marked baseline prolongation of QT/QTc interval (such as QTc intervals repeatedly >450 ms) or any other relevant ECG finding at screening
22. A history of additional risk factors for torsade de pointes (such as heart failure, hypokalemia, or family history of long QT syndrome)
23. Subject was assessed as unsuitable for inclusion by the investigator (e.g., because of inability
    to understand and comply with study requirements or having a condition that would not allow safe participation in the trial)

### Dose escalation

For both trials, the decision to proceed to the next dose group (DG) was based on safety, tolerability, and preliminary PK data (if applicable) in the preceding DGs. The next dose was only given if, in the opinion of the investigator, no safety concerns arose in the preceding DG (i.e., no dose-limiting events occurred) and if none of the pre-specified trial-specific stopping criteria were met. Dose escalation was to be stopped when at least two subjects receiving BI 685509 at one dose level showed relevant individual QT prolongation (absolute QT or QTc >500 ms) or a QTc increase of >60 ms from baseline in connection with an absolute QT >500 ms, confirmed by a repeat ECG recording. Dose escalation was to be stopped if the estimated exposure was expected to exceed a time to peak plasma BI 685509 concentration (C_max_) of 1180 nM and an area under the plasma concentration-time curve over the last 24 hours (AUC_0-24h_) of 14 600 nM·h. Estimation was to be done based on preliminary PK data.

### Further PK endpoints of interest

Renal clearance of BI 685509 from time t_1_ to t_2_ (CL_R,t1–t2_) (2.5 mg ~~dose group [~~DG~~]~~ only) was
a further PK parameter of interest in the single rising dose (SRD) study. Further PK parameters
of interest in the multiple rising dose (MRD) study included the following: steady-state apparent clearance of BI 685509 from plasma (CL/F_ss_), steady-state apparent volume of distribution during
the terminal phase λ_z_ (V_z_/F_ss_), and steady-state renal clearance (CL_R,t1–t2,ss_; for DG1–3 on days 10
and 17); C_max_ after administration at steady state (t_max,ss_); terminal half-life of BI 685509 in plasma (t_½_) (for DGs 1–3 on day 1, after the first dose only); accumulation ratio based on C_max,ss_ (R_A,Cmax_) and AUC_0–τ_ (R_A,AUC_), and linearity index
(for DGs 1–3 on days 10 and 17); and AUC and C_max_ after administration of the *n*th dose
(for DGs 4–5 on day 14).

### ~~Further safety endpoints~~

~~Further evaluation of safety was based on adverse events (AEs; including clinically relevant findings from a physical examination), safety laboratory tests (hematology, clinical chemistry, and urinalysis), 12-lead ECG (CardioSoft EKG System; GE Medical Systems, Freiburg, Germany), vital signs
(BP, heart rate [HR]; Dinamap Pro 100; GE Medical Systems), mean arterial pressure, and
orthostatic testing.~~

~~In the SRD study, orthostatic testing comprised four measurements: in the supine position; immediately after standing up; after 2 min in a standing position; and after 2 min of walking around. In the MRD study, three measurements were performed: in the supine position; immediately after standing up; and after 3 min in a standing position. In the SRD study, orthostatic testing was performed 30 min, 1 h, 2 h, and 3 h after study drug administration. In the MRD study, orthostatic testing was performed on days 1, 10, and 17 at 30 min, 1 h, and 2 h after study drug administration (DG1–3) and on day 1 and day 4 (1 h after morning dose), day 8 (1 h after each dose), and day 14
(at 30 min, 1 h, and 2 h after morning dose) (DG4 and DG5). A baseline measurement was performed in the morning of each trial day with planned orthostatic testing. The term “orthostatic dysregulation” was used to describe symptomatic AEs that occurred during orthostatic testing; typical symptoms of orthostatic dysregulation are dizziness, diaphoresis, tachycardia (HR >100 bpm), and fainting (which
is reflected in the assessment of AE intensity).~~

### Pharmacodynamic assays

*p-VASP/VASP ratio*

For the vasodilatory-stimulated phosphoprotein (VASP) phosphorylation assay in the SRD trial, platelet-rich plasma from whole blood of clinical trial subjects was prepared. In the flow cytometry-based assay, isolated fixed and permeabilized human platelets were incubated with monoclonal rabbit anti-human VASP antibody and monoclonal mouse anti-human phosphorylated (p)-VASP (Ser239) antibody. After incubation, unbound antibodies were removed by washing. In a second step, bound VASP and p-VASP antibodies were detected by anti-rabbit immunoglobulin G (IgG) antibody conjugated with fluorochrome AF488 and anti-mouse IgG antibody conjugated with fluorochrome AF647. After incubation and washing, median fluorescence intensity of AF488 and AF647 was determined by flow cytometry.

*cGMP in plasma and urine*

A level 3 validated liquid chromatography tandem mass spectrometry (LC-MS/MS) assay for the quasi-quantitative analysis of cyclic guanosine monophosphate (cGMP) was employed for the analysis of plasma (SRD and MRD trials) and urine (MRD trial only) samples. cGMP in plasma was analyzed by LC-MS/MS using adequate internal standards. The samples were subjected to automated solid phase extraction in the 96-well plate format followed by reversed-phase LC with gradient elution. The substances were detected and quantified by MS/MS using electrospray ionization in the positive ion mode.

cGMP in urine was analyzed by LC-MS/MS using adequate internal standards. The samples were acidified in the 96-well plate format followed by reversed-phase LC with gradient elution. The substances were detected and quantified by MS/MS using electrospray ionization in the positive ion mode.

~~For the PK analysis and quantification of BI 685509 plasma concentrations in the SRD study, 2.7 mL of blood were taken from an antecubital or forearm vein into a tripotassium ethylenediaminetetraacetic acid (K~~_~~3~~_~~-EDTA) anticoagulant blood-drawing tube at regular intervals for up to 24 h after drug dosing. Urine samples for PK purposes were collected only from subjects in the 2.5 mg DG; a blank urine sample was collected before administration of trial medication, and urine was then collected at time points within 0–4 h, 4–8 h, 8–12 h, and 12–24 h of drug dosing. BI 685509 concentrations in plasma and urine were determined by a validated liquid chromatography tandem mass spectrometry (LC-MS/MS) assay. For the pharmacodynamic analysis and quantification
of vasodilatory-stimulated phosphoprotein (VASP) and phosphorylated (p)-VASP in platelets,
5.0 mL of blood were taken from an antecubital or forearm vein into a trisodium citrate anticoagulant
blood-drawing tube at regular intervals for up to 8 h after drug dosing~~. ~~For quantification of cGMP in plasma, 2.7 mL of blood were taken into a K~~_~~3~~_~~-EDTA anticoagulant blood-drawing tube~~. ~~cGMP levels in plasma and urine were considered as exploratory biomarkers, and the LC-MS/MS assays used were validated accordingly. The p-VASP/VASP ratio in platelets was also considered to be an exploratory biomarker, and the fluorescence-activated cell sorting assay employed to measure VASP and p-VASP was validated accordingly.~~

~~For quantification of BI 685509 plasma concentrations in the MRD study, 2.7 mL of blood
were taken from an antecubital or forearm vein into a K~~_~~3~~_~~-EDTA anticoagulant blood-drawing tube
at prespecified sampling times over 20 days. Urine voided during the sampling intervals (days 1–4, 10–11, and 17–20) was collected. Plasma and urine concentrations of BI 685509 were determined
by validated LC-MS/MS assays. For analysis of plasma cGMP concentrations, 2.7 mL of blood were taken from an antecubital or forearm vein into a K~~_~~3~~_~~-EDTA anticoagulant blood-drawing tube at prespecified times on days 1, 10–11, and 17. cGMP levels in plasma and urine were considered exploratory biomarkers, and the LC-MS/MS methods used were validated accordingly.~~

~~PK parameters were calculated by noncompartmental analysis using Phoenix® WinNonlin® software (version 6.3; Pharsight Corporation, Mountain View, CA, USA).~~

### Assessment of dose proportionality and linearity

Dose proportionality was evaluated using a linear regression model applied to log-transformed data.
A two-sided 95% confidence interval for the slope (β) was computed; perfect dose proportionality corresponded to a slope of 1. Dose proportionality was assessed for C_max_, AUC_0–tz_, and AUC_0–∞_ (DG1–3, day 1); for AUC_0–24,ss,8_ and C_max,ss,8_ (DG1–3, day 10); and for AUC_0–24,ss,15_ and C_max,ss,15_ (DG1, day 17). The linearity index was calculated based on AUC_τ,ss_ (DG1–3, day 10; and DG2–3,
day 17) and AUC_0–∞_ in the MRD study.

**Supplementary Table S1** Statistical analysis of dose proportionality of BI 685509 after single and multiple oral administration: MRD study

| **Parameter** | **BI 685509 dose** | ***n*** | **β** | **SE (β)** | **95% CI (β)** |
| --- | --- | --- | --- | --- | --- |
| C_max_, nmol L^–1^ | 0.5 mg qd, 2.5 mg qd, 5.0 mg qd^a^ | 27 | 0.9521 | 0.0658 | 0.8167–1.0876 |
| AUC_0–tz_, nmol h L^–1^ | 0.5 mg qd, 2.5 mg qd, 5.0 mg qd^a^ | 27 | 0.9077 | 0.0700 | 0.7635–1.0520 |
| AUC_0–∞_, nmol h L^–1^ | 0.5 mg qd, 2.5 mg qd, 5.0 mg qd^a^ | 27 | 0.8840 | 0.0698 | 0.7403–1.0277 |
| AUC_0–24,ss,8_,^b^ nmol h L^–1^ | 0.5 mg qd, 2.5 mg qd, 5.0 mg qd^c^ | 26 | 0.8670 | 0.0439 | 0.7764–0.9576 |
| C_max,ss,8_,^b^ nmol L^–1^ | 0.5 mg qd, 2.5 mg qd, 5.0 mg qd^c^ | 26 | 0.9038 | 0.0487 | 0.8033–1.0043 |
| AUC_0–24,ss_, nmol h L^–1^ | 0.5 mg qd, 1.0 mg qd, 2.5 mg qd, 5.0 mg qd^d^ | 35 | 0.8460 | 0.0425 | 0.7595–0.9324 |
| C_max,ss_, nmol L^–1^ | 0.5 mg qd, 1.0 mg qd, 2.5 mg qd, 5.0 mg qd^d^ | 35 | 0.8820 | 0.0460 | 0.7884–0.9755 |

^a^After the first dose (day 1) in DG1–3.

^b^Subscript numbers (e.g., C_max,ss,8_) represent the *n*th dose.

^c^After the dose (day 10) in DG1–3.

^d^After the dose (day 10) in DG1–3 and after the dose (day 17) in DG1.

β, slope parameter; AUC, area under the plasma concentration–time curve; AUC_0–tz_, AUC of BI 685509 in plasma from time 0 to the last quantifiable data point; CI, confidence interval; C_max_, peak plasma concentration; DG, dose group; MRD, multiple rising dose; qd, once daily; SE, standard error; ss, steady state.

**Supplementary Table S2** Placebo-corrected mean changes from baseline in BP: MRD study

| Dose group | Time post dose (h) | DG1 | DG2 | DG3 | DG4 | DG5 |
| --- | --- | --- | --- | --- | --- | --- |
| Dosage^a,b,c^ |  | 0.5, 1.0 mg qd | 2.5 mg qd, bid | 5.0 mg qd | 1.0, 2.0, 3.0 mg tid | 1.0, 2.0, 4.0 mg tid |
| **Systolic BP, mmHg** |  |  |  |  |  |  |
| Trial day 1 | 0.5 | –5.59^d^ | –9.36^d^ | –5.51^d^ | NA | NA |
|  | 1.0 | –10.15^d^ | –11.02^d^ | –10.62^d^ | NA | NA |
|  | 2.0 | –5.15 | –9.13^d^ | –5.40 | NA | NA |
| Last dosing day | 0.5 | –3.26 | –0.30 | –5.15 | –7.80^d^ | –6.98^d^ |
|  | 1.0 | –3.49 | –0.97 | –4.09 | –9.13^d^ | –6.64 |
|  | 2.0 | –3.38 | –1.30 | –6.98^d^ | –3.41 | –4.37 |
| **Diastolic BP, mmHg** |  |  |  |  |  |  |
| Trial day 1 | 0.5 | –3.31 | –6.67^d^ | –4.52^d^ | NA | NA |
|  | 1.0 | –2.20 | –7.11^d^ | –6.18^d^ | NA | NA |
|  | 2.0 | –1.86 | –6.33^d^ | –5.74 | NA | NA |
| Last dosing day | 0.5 | –4.07^d^ | –0.34 | –2.81 | –6.32^d^ | –6.30^d^ |
|  | 1.0 | –2.18 | –1.56 | –6.67^d^ | –6.93^d^ | –7.25^d^ |
|  | 2.0 | –5.07^d^ | –4.45 | –8.50^d^ | –3.88 | –3.64 |
| **Mean arterial pressure, mmHg** |  |  |  |  |  |  |
| Trial day 1 | 0.5 | –3.96^d^ | –7.51^d^ | –4.78^d^ | NA | NA |
|  | 1.0 | –4.74^d^ | –8.36^d^ | –7.60^d^ | NA | NA |
|  | 2.0 | –2.85 | –7.21^d^ | –5.56^d^ | NA | NA |

| Last dosing day | 0.5 | –3.80^d^ | –0.28 | –3.88 | –6.85^d^ | –6.36^d^ |
| --- | --- | --- | --- | --- | --- | --- |
|  | 1.0 | –2.62 | –1.32 | –6.11^d^ | –7.71^d^ | –6.88^d^ |
|  | 2.0 | –4.51^d^ | –3.36 | –8.29^d^ | –3.76 | –3.71 |

^a^Dosage uptitration schedule.

^b^Data shown are for the treated set (*n* = 9 subjects per DG).

^c^Times post dose relate to the morning drug administration on the trial day indicated.

^d^The 90% confidence interval did not include zero.

bid, twice daily; BP, blood pressure; DG, dose group; MRD, multiple rising dose; NA, not available; qd, once daily; tid, three times daily.

**Supplementary Fig. S1** Adjusted means from repeated measures analysis of placebo-corrected percentage of baseline of p-VASP/VASP ratio in platelets after single oral administration of BI 685509 by treatment (SRD trial)


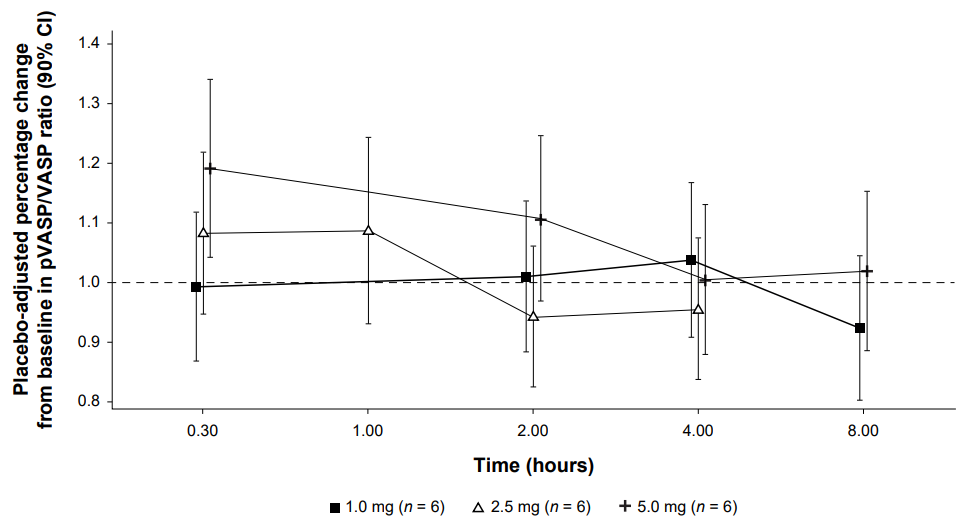


**Supplementary Fig. S2** Adjusted means from repeated measures analysis of placebo-corrected percentage of baseline of cGMP in plasma after single oral administration of BI 685509 by treatment (SRD trial)


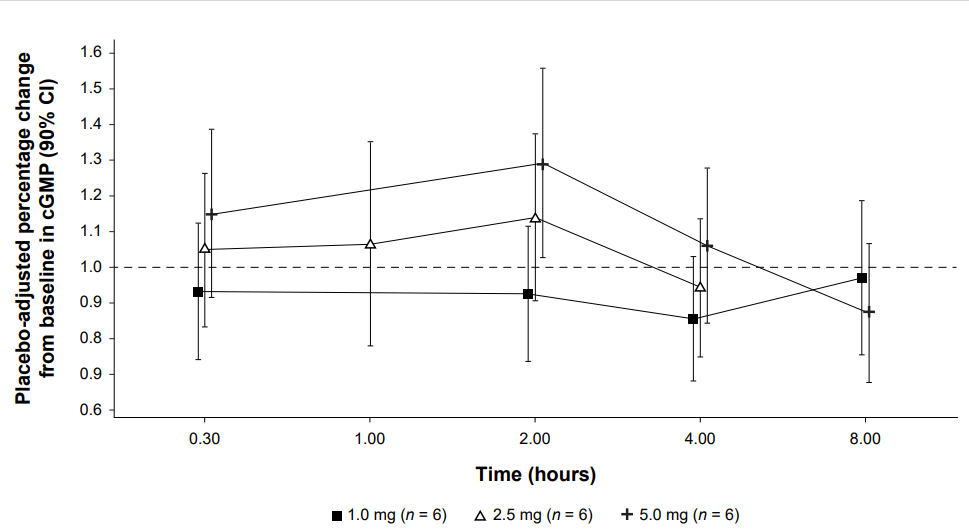


**Supplementary Fig. S3** Arithmetic mean (±SD) baseline-corrected cGMP percentage–time profiles in plasma after single and multiple oral administration of placebo or BI 685509 by treatment (DG 1–3) (MRD trial)


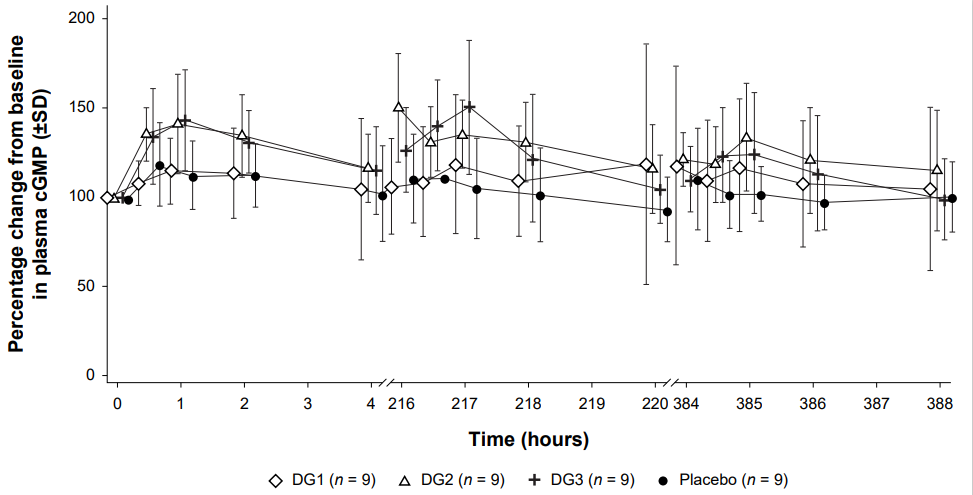


**Supplementary Fig. S4** Arithmetic mean (±SD) baseline-corrected cGMP by creatinine-–time profiles in urine after single and multiple oral administration of placebo or BI 685509 by treatment (DG 1–3) (MRD trial)


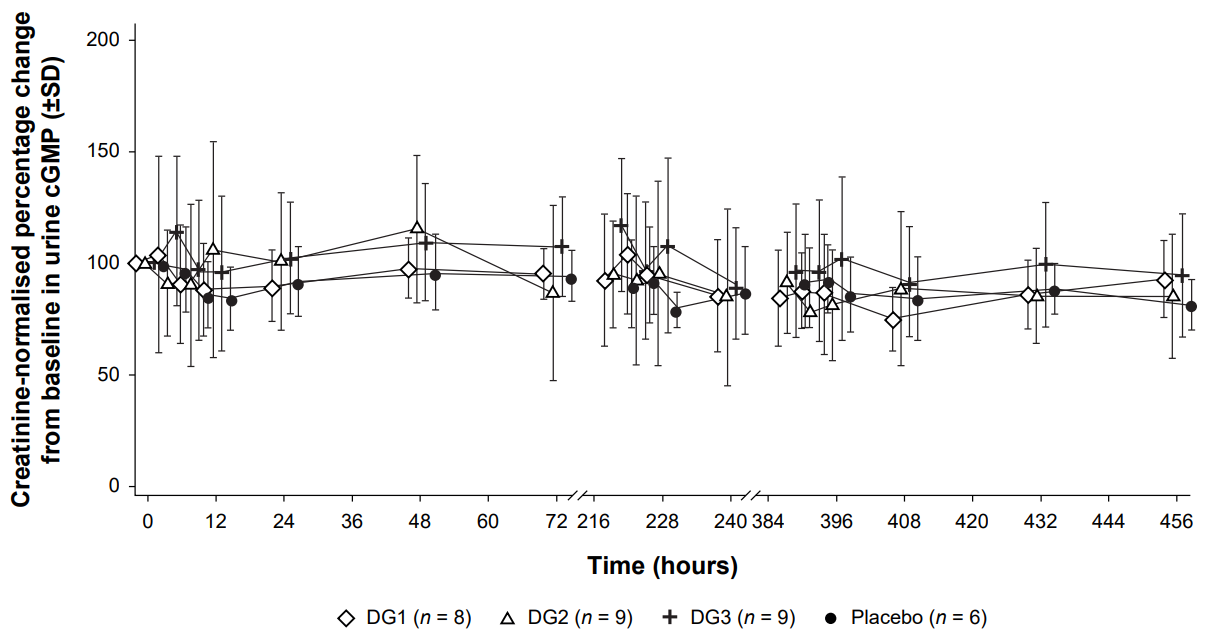

Supplement: Supplementary file 1 — Supplementary file1 (DOCX 360 KB) [file 210_2024_3165_MOESM1_ESM.docx]
